# Can elderly patients regain their preoperative functional level after distal radius fracture type A? Results from a fracture register study using PROM

**DOI:** 10.3389/fsurg.2023.877252

**Published:** 2023-04-05

**Authors:** F. Von Matthey, J. Rammensee, M. Müller, P. Biberthaler, H. Abel

**Affiliations:** Department of Trauma Surgery, Klinikum rechts der Isar, Technische Universität München, Munich, Germany

**Keywords:** PROM, distal radius fracture type A, outcome, Munich Wrist Questionnaire, age

## Abstract

**Introduction:**

Although distal radius fractures (DRFs) are the most common fractures of the human body, there are still ongoing debates concerning the treatment for type A fractures, especially in elderly patients. In spite of good clinical outcomes, it remains unclear whether elderly patients, especially, could regain the preoperative functional level of the wrist. Therefore, we have quantified wrist function within a retrospective study design using patient-reported outcome measures (PROM) and we have analyzed the influence of age between control and patient collective and young vs. old, respectively.

**Patients and methods:**

The retrospective study included all patients with a surgically treated DRF type A and a control group of healthy patients, age and gender matched. The function of the wrist was examined by using a self-assessment questionnaire called the Munich Wrist Questionnaire (MWQ) according to the patient-related outcome measurements PROM.

**Results:**

We could enroll 110 patients and controls, and the average follow-up was 66 months. Subgroup matching induced similar age group distribution: in both groups, 7 individuals <30 years, 67 between 31 and 64 years, 29 between 65 and 79 years, and 7 individuals >80 years, were enrolled, respectively. In the fracture group, women were significantly older than men (59 ± 15 vs. 47 ± 17 (*M* ± SD). There was no significant difference between the control and the patient groups (96 ± 6 vs. 95 ± 7). The function was significantly different between controls and patients <30 years (100 ± 1 vs. 98 ± 2). In the control group, there was a functional difference in the age group <30 compared with 65–79 and >80 and in the age group 30–64 compared with 65–79 and >80. In the control group, the function was found to be significantly decreasing with advancing age, whereas in the patient group, this influence was absent. A correlation analysis showed a worse function with increasing age in the control group and therefore a negative correlation. In the fracture group, a similar result could not be obtained.

**Discussion:**

Age has a relevant influence on wrist function. Although the wrist function decreases significantly with aging, in the patient group, this influence is absent, and the functional results after surgery are excellent. Even elderly patients can regain their preoperative functional level.

## Introduction

Distal radius fracture (DRF) is a frequently occurring injury (1–3). Although there are several classification systems, the AO classification is the one that is most widely distributed. While therapeutic guidelines for B- and C-type fractures are relatively clear, for A-type fractures, there is an ongoing debate in scientific literature about the optimal therapeutic strategy to be adopted. On this count, the therapeutic options vary from simple closed reduction and cast up to open reduction and internal fixation (ORIF) for dislocated A3 fractures. In this respect, many prospective randomized trials were not able to identify a significant difference between the different therapeutic branches (3–6).

While results from Swedish fracture registers suggest a more conservative treatment (7, 8), German surgeons seem more willing to opt for surgery (3, 9). Surgery allows an early start of wrist movement, while casting for 6 weeks is accompanied by muscle loss and often loss of autarky for the elderly. Several studies could show good to excellent results after surgical treatment even of the super elderly aged over 80 years (6, 10, 11). However, all studies published so far have analyzed the actual outcome after surgery without paying attention to the age-related wrist function. To our knowledge, no study exists that analyzes the functional level of the elderly after surgery compared with the healthy elderly wrist. This is of special interest because it is questionable whether elderly patients regain their preoperative function level. Age or the aging process could be a significant factor in convalescence, and functional results in the elderly after surgery could be worse than that in younger patients, but the results may be excellent compared with a healthy control of the same age.

Therefore, the present study has developed a fracture register and has analyzed the outcome of distal radius fractures type A by using the Munich Wrist Questionnaire (MWQ) in accordance with a patient-reported outcome measures (PROM), which has been validated and published previously (12).

Therefore, the aims of this study are to:

(1) Quantify wrist function within a retrospective study design using a PROM on patients enlisted in our fracture register and suffering from AO type A-fracture/surgical therapy ORIF.
(2) Compare the obtained data with age and matched pair controls in respect of relevant age groups.

## Patients and methods

This retrospective cohort study was approved by the local ethics committee (409/15 s), and all control individuals as well as patients gave their written informed consent prior to participation.

### Study group

For this retrospective setting, we identified patients from our fracture register who were suffering from distal radius fractures, classified as A-type fractures according to the AO classification system, and who were treated surgically within an observation period from 2006 until 2016.

The exclusion criteria were external fixation, primary surgery elsewhere, history of previous trauma, or multiple injuries.

### Control collective

For control, healthy individuals were recruited and demographic data such as age and gender were adjusted to the study collective in order to obtain an objective control collective to the greatest extent possible in terms of matched pairs. The exclusion criteria were a history of previous injuries of the upper extremities and relevant diseases with a potential negative influence of wrist function, such as rheumatoid arthritis.

### Surgical therapy

All patients were operated by employing the following procedure: the standardized modified approach described by Henry and volar plating using either a monoaxial or polyaxial locking plate (i.e., Depuy Synthes, Medartis, etc.).

Indications for the surgery were a displaced, instable fracture and unsatisfactory or instable closed reduction. No ulnar styloid fracture had to be repaired surgically. After surgery, a dorsal splint was applied for 2 weeks, and movement without weight bearing was allowed immediately. All patients received a standardized after-care physiotherapy program and visited our outpatient center for regular checkups.

### Evaluation of wrist function

For functional measurement of wrist function, all control individuals as well as all patients filled in the standardized MWQ.

This questionnaire was designed as a scientific instrument to analyze wrist function according to a PROM. This instrument allows for a quantitative measurement of wrist function and provides results as a percentage of a potential 100% function; it has been validated previously (12).

The MWQ is a self-administered questionnaire, developed to evaluate the symptoms and physical function of the wrist. It has two components: the main disability/symptom section and the range of motion (ROM) sections. The main component of the MWQ is a 13-item scale concerning pain (5), work and activities of daily living (7), and grip strength (1). In the second part of the MWQ, the ROM is assessed (dorsal extension, dorsal flexion, supination and pronation, and ulnar and radial deviation) by using drafts.

### Analysis

Epidemiological and demographic data were summarized and given in mean ± standard deviation (*M* ± SD) for age and postoperative observation period. Frequencies of gender, etc., were given in (%). For subgroup analysis, the functional results were summarized by calculating mean values and standard deviation (*M* ± SD).

Control individuals as well as patients filled in the MWQ and the results were computed to a relative quantitative functional value given in % from the standardized 100% function of the questionnaire.

When examining the analysis by considering the corresponding age groups, a primary distinction was made between young and old patients (<65 and >64 years). For quantitative analysis, the wrist function parameters of the control and study groups were compared using the Mann–Whitney *U*-test. To analyze the influence of age, control and study collective were divided into four subgroups: <30, 30–64, 65–79, and >80 years. Every subgroup was compared between the fracture and control groups. In a further step, within each control and fracture group, the functions of these subgroups were analyzed by using Kruskal–Wallis one-way analysis of variances (ANOVA). After identifying significant differences, as a *post hoc* test, Dunn's method for pairwise comparison was used to identify significant differences between the groups. Furthermore, we analyzed the age and MWQ values of both control and fracture groups using the Pearson and Spearman test to find a possible correlation. In this regard, a *p*-value <0.05 was considered statistically significant.

## Results

### Epidemiological and demographic data

#### Study group

In this study, 110 patients suffering from A-type fractures of their distal radius were enrolled. All of them were treated by employing the ORIF strategy using volar plating with locking plates. The epidemiological and demographic data are given in Table 1.

**Table 1:** Epidemiological and demographic data of control and study group.

	Age (MW ± SD)	Female, *n* (%)	Male, *n* (%)	Dominant hand, *n* (%)	Non-dominant hand, *n* (%)
Control	55 ± 19	71 (65)	39 (35)	55 (50)	55 (50)
Fracture	56 ± 16	88 (80)	22 (20)	47 (43)	63 (57)

The mean follow-up time was 66 ± 29 months (range 8–116 months).

#### Control group

From these patients, we identified control individuals of similar age and sex and without relevant pathology in the upper extremity, such as trauma and rheumatoid arthritis. The epidemiological and demographic data are given in Table 1. In the control group, due to a matched paired study design, 110 individuals were additionally enrolled and subgroup matching induced similar age group distribution.

### Age and gender

#### Study group

The average age of patients with type A fracture was 56 ± 16 years.

Equivalent to the subgroup analyses: 7 individuals <30 years, 67 individuals between 31 and 64 years, 29 individuals between 65 and 79 years, and 7 individuals >80 years, respectively (Table 1).

There was a total of 88 female and 22 male patients in the fracture group and women were significantly older than men (59 ± 15 vs. 47 ± 17 years; *p* = 0.006).

#### Control group

The average age of the control group was 55 ± 19 years.

In the control group, due to a matched paired study design, 110 individuals were additionally enrolled and subgroup matching induced similar age group distribution. Hence, there were 7 control individuals <30 years, 67 control individuals between 31 and 64 years, 29 control individuals between 65 and 79 years, and 7 control individuals >80 years, respectively (Table 1).

There was a total of 71 female and 39 male patients in the control group. Female and male controls showed no significant difference in age (56 ± 20 vs. 53 ± 16; *p* = 0.296).

### Functional results in the MWQ

Control individuals as well as patients filled in the MWQ and the results were computed to a relative quantitative functional value given in % from the standardized 100% function of the questionnaire. The results are given in Table 2.

**Table 2:** This table depicts the quantitative functional results of the wrist joint according to a standardized patient-reported outcome measures (PROM) instrument.

MWQ in (%) MW ± SD	All	<30, *n* = 7	30–64, *n* = 67	65–79, *n* = 29	>80, *n* = 7
Control	96 ± 6	100 ± 1	97 ± 4*	93 ± 7^#^	89 ± 7
Fracture	95 ± 7 ŧ	98 ± 2	95 ± 7	93 ± 8	95 ± 9

In the overall analysis, no significant difference was calculated. There was a significant difference in the control group: ANOVA on ranks: *p* < 0.001; Dunn's pairwise: <30 vs. 65–79 (*p = 0.001), <30 vs. >80 (*p < 0.001), 30–64 vs. 65–79 (^#^*p* < 0.001), and 30–64 vs. >80 years (^#^*p* = 0.003). There was no significant difference in the fracture group (*p* = 0.54).

**Figure 1:**
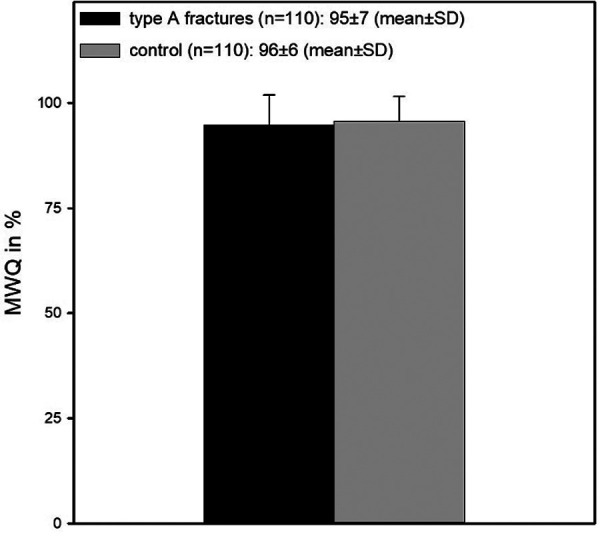
This figure shows MWQ values for both control and fracture groups. There was no significant difference (96 ± 6 vs. 95 ± 7; *p* = 0,144).

In the overall analysis, there was no significant difference between the control and patient groups (96 ± 6 vs. 95 ± 7; *p* = 0,144) (Figure 1).

When viewing the analysis from the perspective of corresponding age groups, a primary distinction was found between young and old patients.

**Figure 2:**
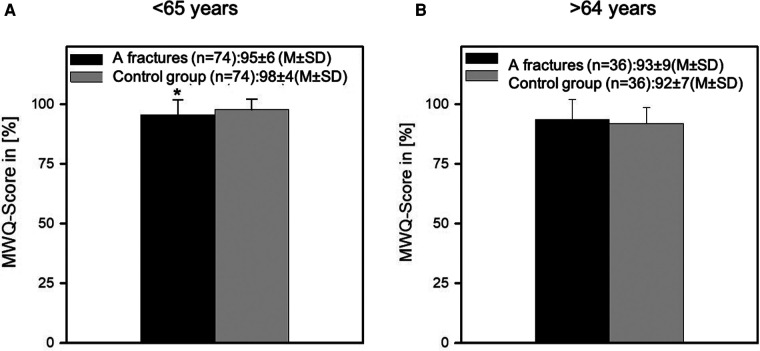
This figure depicts the MWQ of the young patients (<65 years) (**A**) and the elderly patients (>64 years) (**B**) compared with their matched control group. While the younger patients could not reach the functional level of the control group (98 ± 4 vs. 95 ± 6, *p* < 0.001), the function of the old patients >64 years showed no significant difference (92 ± 7 vs. 93 ± 9; *p* = 0.054).

For this purpose, the function of patients <65 years was compared with that of the corresponding controls. The control group showed a significantly better function (98 ± 4 vs. 95 ± 6, *p* < 0.001), whereas the function of old patients >64 years showed no significant difference in function (92 ± 7 vs. 93 ± 9; *p* = 0.054) (Figure 2).

To investigate this result further, the function of the age groups <30, 30–64, 65–79, and >80 years was examined (Table 2).

There was no difference in the function between control group and postoperative patients who were <30 years of age (100 ± 1 vs. 98 ± 2; *p* = 0.0585), whereas the control patients of the 30–64 age group showed a significantly better wrist function than the fracture group patients (97 ± 4 vs. 95 ± 7; *p* = 0.005).

In the age groups >65–79 years (93 ± 7 vs. 93 ± 8; *p* = 0.308) and >80 years (89 ± 7 vs. 95 ± 9; *p* = 0.073), there was no functional difference.

### Function between age groups

In order to further investigate the function in the course of aging, the function within the different age groups was compared in both the control and the fracture groups.

**Figure 3:**
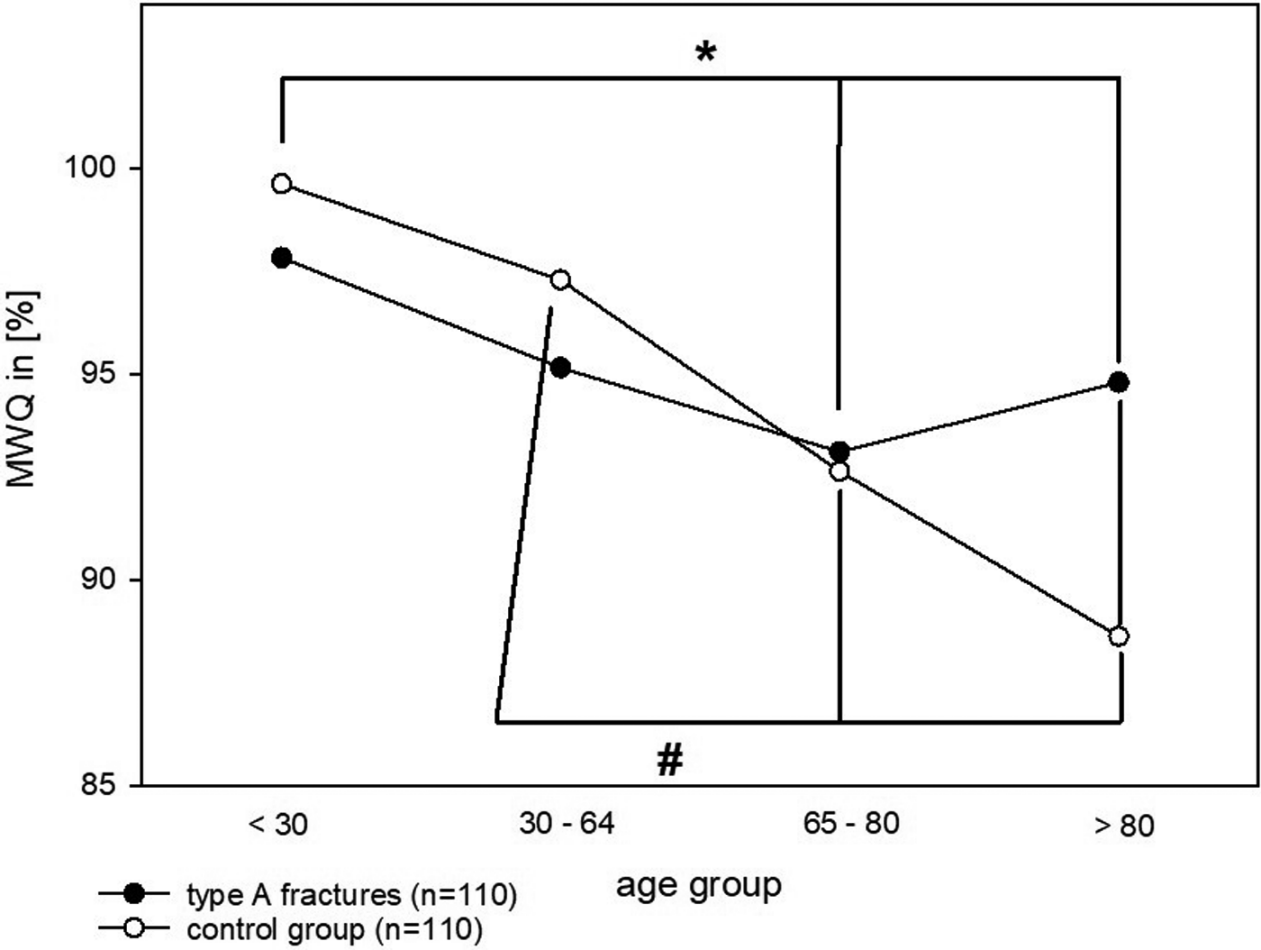
This figure shows MWQ values for both control and fracture groups separated in their age groups. There was a significant difference in the control group: ANOVA on ranks: *p* < 0.001; Dunn's pairwise: <30 vs. 65–79 (*p = 0.001), <30 vs. >80 (*p < 0.001), 30–64 vs. 65–79 (#*p* < 0.001), and 30–64 vs. >80 years (#*p* = 0.003). There was no significant difference in the fracture group (*p* = 0.54).

Here, with a *p*-value of 0.54, there was no statistically significant difference in function within the different age groups of the fracture group. In contrast, the function within the control group differed significantly between the different age groups (*p* < 0.001) (Table 2, Figure 3).

In further examination of these results, ANOVA on ranks revealed a significant difference in the age groups between <30 and 65–79 (*p* = 0.001), <30 and >80 (*p* < 0.001), 30–64 years and 65–79 (*p* < 0.001) as well as between 30–64 and >80 years (*p* = 0.003).

Interestingly, in the control group, the function was significantly decreasing with advancing age, whereas in the patient group, this influence was absent (Figure 3). This was also confirmed by the Pearson and Spearman correlation test (fracture: *p* > 0.05; control: negative correlation −0.534/−0.583, *p* < 0.001).

## Discussion

In this study, we could demonstrate that the factor of age is critical for wrist function.

Patients suffering from a distal radius fracture type A, enrolled in a fracture register within a retrospective cohort design, were analyzed. This allowed us to enroll as many patients as possible overseeing the longest possible observation period. This is in line with previous published studies that also used retrospective designs (8, 13–15).

Moreover, the presented study design, a fracture register within a retrospective cohort design, has several advantages over a randomized controlled trial, particularly with regard to the elimination of the selection bias of patients and surgeons. Moreover, PROM is a suitable tool for fast and easy data collection. The outcome of the control group significantly degenerated with age, whereas the outcome of the fracture group remained comparably good regardless of age. This result should be taken into consideration when deciding about surgical options for elderly patients.

Although there are many PROMs like the DASH (16, 17) or the PRWE (18, 19), we decided to use the Munich Wrist Questionnaire prepared by Beirer et al. (12), which focuses on the wrist and includes not only objective but also subjective parameters.

The MWQ is a relatively novel PROM. Accordingly, not many publications exist compared with the DASH or PRWE, for example. These two PROM questionnaires contain item scales concerning the patient's health status for the preceding week, such as the degree of difficulty in performing certain physical activities, the severity of pain, activity-related pain, tingling, weakness, and stiffness and the effect of the restricted upper limb on social activities, work, sleep, and self-image. The MWQ also consists of questions concerning daily activities, pain, etc., but it seeks to determine the patients’ ROM with a draft of the possible ROM. This questionnaire is more suitable for wrist fractures to detect the limitations of the ROM as failed wrist movement cannot be compensated by shoulder movement, whereas the daily activities tested in the DASH can be compensated by other means (20, 21).

This study does not take the radiological outcome into account because a good radiological result does not necessarily accompany a good clinical outcome (22, 23).

From our fracture register, we were able to include 110 patients with distal radius fractures in our study, from whom a complete data set was available. The number of patients corresponded to that of comparable studies (4, 9, 24, 25).

In order to establish a good comparison with the wrist function of the healthy normal population, a collective of volunteers without existing or pre-existing disease in the area of the upper extremity served as a control group. This is where our study differs from other studies, such as Ju et al. (6), which focus on the comparison of conservative and operative treatment. However, our study attempts to focus on surgically treated distal radius fractures; therefore, the control group consists of healthy volunteers.

In the non-differentiated comparison between the fracture and the control groups, there was no significant difference in function between the two groups (96 ± 6 vs. 95 ± 7) (Figure 1). We consider this result to be open for critical discussion, and this, in our opinion, can be most likely attributed to the low number of patients in this study.

A limitation of this study is the small patient number, although comparable to other studies as mentioned above. Unfortunately, only 110 patients with a complete data set could be enrolled. In terms of subgroup analysis, the patient number is again small. Therefore, the results should be confirmed with a larger patient collective.

The follow-up duration of this study was 5.5 years (range 8–116 months). This range from 8 months up to nearly 10 years warrants critical discussion, because this range results in patients having heterogenous baselines and the results might be biased. There are several studies with a short-term follow-up of only 1 year, but there are also authors who have analyzed data from up to 12 or 20 years (26, 27). However, the medium-term and long-term results seem relevant and important, as Landgren et al. e.g., could show that patient function, analyzed with the DASH score, improved significantly from the 2- to the 12-year follow-up period (27). Nevertheless, the outcomes in the present study do have a minimal standard deviation in spite of the wide range of the follow-up points. Therefore, we think this bias can be disregarded.

Although the overall postoperative function is very good, we were able to show in the further analysis of the data that the function level of the young patients (<65 years) was significantly worse in comparison with the corresponding control group (98 ± 4 vs. 95 ± 6; *p* < 0.001), whereas the function level of the old patients showed no significant difference (92 ± 7 vs. 93 ± 9; *p* = 0.054) compared with the corresponding controls (>64 years). As this result seems slightly unplausible at first sight because it is unlikely that the elderly and super-elderly patients will have better functional outcomes after surgery compared with the healthy control younger patients, we performed an in-depth investigation of the function level of the control group. This revealed an age-dependent worsening of the wrist function in the course of aging. This seems logical when one considers that the wrist is also affected by signs of aging (age-related diseases) such as arthrosis, etc. and is also a joint that is primarily affected by osteoporosis, even in the absence of a fracture.

Therefore, it can be stated that even elderly patients (>64 years) have a good functional outcome after surgery of a distal radius fracture type A according to the AO classification. Moreover, the natural worsening of the wrist function should be taken into consideration whenever functional results are discussed.

Hence, we strongly recommend for further studies on distal radius fractures to divide patient collective at least in a group below and a group above 65 years, similar to studies on proximal femur fractures. This is supported by other authors (28). This comes along with the studies published by Tulipan et al. who could show that the elderly also had good outcomes. They stated that osteosynthesis should be offered to the elderly (10, 29). However, most of these studies deal with all types of distal radius fractures and/or analyze the outcomes after surgery without paying attention to the healthy control group. Another aspect is the comparison with the conservatively treated patients, which is of significant interest and answers another question.

However, a comparison with the conservatively treated patients suffering from a distal radius fracture type A would be very interesting as well and is being planned for inclusion in the fracture register as part of an expansion exercise.

In this study, we could primarily show that age has a relevant influence on wrist function.

Furthermore, the natural loss of wrist function with age could be the reason that the difference in function between patients with distal radius fractures and the control group patients no longer exists in old age. Ultimately, this means that elderly patients, in particular, benefit from surgical fracture treatment—including extra-articular distal radius fractures—as they can regain their initial functional level postoperatively.

## Conclusion

This study shows that age has a relevant influence on wrist function. Although the wrist function decreases significantly with aging, in the patient group, this influence was absent, and the functional results after surgery were excellent. Patients could regain their preoperative level of function. This is of great significance with regard to the question whether the DRF type A should be treated surgically.

## Data Availability

The raw data supporting the conclusions of this article will be made available by the authors without undue reservation.
